# Exosomes as Drug Delivery Systems: Endogenous Nanovehicles for Treatment of Systemic Lupus Erythematosus

**DOI:** 10.3390/pharmaceutics13010003

**Published:** 2020-12-22

**Authors:** Ana Ortega, Olga Martinez-Arroyo, Maria J. Forner, Raquel Cortes

**Affiliations:** 1 Cardiometabolic and Renal Risk Research Group, INCLIVA Biomedical Research Institute, 46010 Valencia, Spain; aortega@incliva.es (A.O.); omartinez@incliva.es (O.M.-A.); Maria.Jose.Forner@uv.es (M.J.F.); 2 Internal Medicine Unit, Hospital Clinico Universitario, 46010 Valencia, Spain

**Keywords:** extracellular vesicles, exosomes, microparticles, drug delivery, therapy, autoimmunity, systemic lupus erythematosus

## Abstract

Exosomes, nanometer-sized lipid-bilayer-enclosed extracellular vesicles (EVs), have attracted increasing attention due to their inherent ability to shuttle proteins, lipids and genes between cells and their natural affinity to target cells. Their intrinsic features such as stability, biocompatibility, low immunogenicity and ability to overcome biological barriers, have prompted interest in using exosomes as drug delivery vehicles, especially for gene therapy. Evidence indicates that exosomes play roles in both immune stimulation and tolerance, regulating immune signaling and inflammation. To date, exosome-based nanocarriers delivering small molecule drugs have been developed to treat many prevalent autoimmune diseases. This review highlights the key features of exosomes as drug delivery vehicles, such as therapeutic cargo, use of targeting peptide, loading method and administration route with a broad focus. In addition, we outline the current state of evidence in the field of exosome-based drug delivery systems in systemic lupus erythematosus (SLE), evaluating exosomes derived from various cell types and engineered exosomes.

## 1. Introduction

Autoimmune diseases are among the leading causes of morbidity and mortality associated with chronic disease worldwide, especially in women, who comprise more than 90% of affected patients [1,2]. Autoimmune diseases are classified in two types, the first of which is organ-specific and the second systemic, wherein the immune response attacks different organs and tissues simultaneously, as exemplified by diseases such as systemic lupus erythematosus (SLE) [3]. The pathogenesis of SLE hinges on loss of tolerance and sustained autoantibody production, characterized by the presence of autoreactive T cells and hyperactive B cells which produce autoantibodies; these later form immune complex deposits that damage different tissues on which autoantigens are expressed [3,4,5]. Although non-steroidal anti-inflammatory drugs such as glucocorticoids (GCs) and immunosuppressive therapies are often administered to SLE patients to relieve immunological inflammation during disease progression [6,7,8], these drugs can cause serious side effects due to their toxicity and the lack of target tissue disease. Several investigations point to cell-based therapies, such as stem cell transplantation, to treat SLE [9,10,11], but these are expensive and add to the long-term medical costs associated with the disease. These drawbacks underscore the urgent need to identify safe and effective therapies for SLE prevention and treatment. The development of drug delivery vehicles has recently emerged as a novel therapeutic approach in autoimmune diseases [12,13,14], being exosomes one of the most promising nanocarriers due to their various therapeutic advantages [15,16,17].

Exosomes are lipid-bilayer-enclosed extracellular vesicles released by many cell types in both normal and pathological conditions, and which transport nucleic acids, lipids and proteins between cells [18,19]. As drug and gene delivery carriers, exosomes have unique advantageous characteristics including encapsulating endogenous bioactive molecules, low immunogenicity, biodegradability, longer circulation time, smaller size than other nanocarriers and the ability to cross many biological barriers [15,17]. 

Current evidence shows that exosomes are able to modulate the immune system, as shuttles for antigen presentation [20,21,22,23], as immunosuppressive effectors of inflammation process and as immunologic agents for immunotherapy, emerging as promising tools for therapeutic delivery in autoimmune diseases [24,25]. In SLE, many studies have demonstrated that circulating exosomes are immunologically active and their levels correlate with disease activity [26,27]. In addition, through analysis of exosomal-derived microRNAs (miRNAs) levels, distinct miRNA have been identified to discriminate lupus nephritis (LN) [28], as predictors of early fibrosis [29,30,31], and response to therapy in LN [32]. Therefore, exosomes serve as novel biomarkers and predictors of SLE progress. 

Due to their suitable proprieties, as well as their known therapeutic effects, exosomes are attracting the curiosity of researchers to develop exosome-based drug delivery systems. This review centers first on the biogenesis of natural exosomes and their function in SLE. Next, we will narrow our focus to identify the key components enabling successful exosome-based drug delivery, to unravel the potential of exosomes for disease treatment, finally covering new insights in exosome-based drug and gene delivery for future clinical use in SLE. 

## 2. Biogenesis and Function of Extracellular Vesicles

Extracellular vesicles are small spherical lipid bilayer-coated vesicles, secreted by multiple cell types and present in many body fluids, which mediate intercellular communication [33,34]. Nowadays, interest in extracellular vesicles (EVs) centers mainly on their functions as component exchangers and as signaling transmitters under both normal and pathological conditions [35]. EVs display heterogeneity between different subtypes and are classified according to their size (small or large EVs), composition (CD63+/CD81+, cargos) or biogenesis (exosomes, microvesicles and apoptotic bodies) [18,33,34].

**Figure 1:**
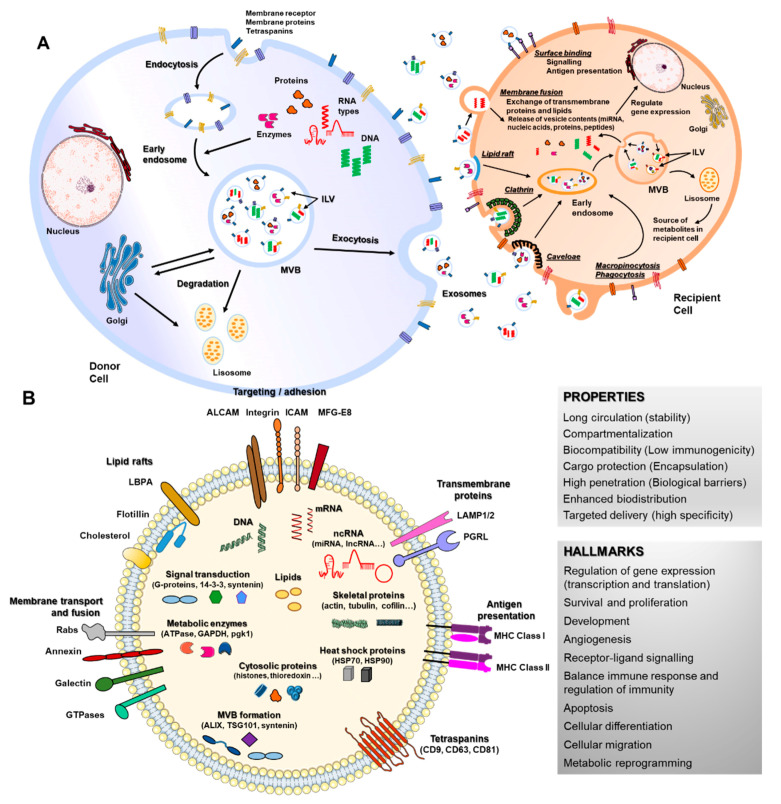
Biogenesis, secretion, uptake and molecular composition of exosomes. (**A**) exosomes from intraluminal vesicles (ILVs) in multivesicular bodies (MVBs) are secreted extracellularly by fusion with the cellular membrane. Next, exosomes interacting with recipient cells directly by surface binding, membrane fusion or internalization will target exogenous exosomes in the canonical endosomal pathway by lipid raft, clathrin, caveloae or micropinocytosis/phagocytosis processes. (**B**) exosomes can contain different types of cell surface proteins (tetraspanins, integrins, major histocompatibility complex (MHC), etc.), intracellular protein (skeletal proteins, heat shock proteins, etc.), nucleic acids (RNA, DNA, microRNAs (miRNA), long non-coding RNA (lncRNA), etc.), amino acids, lipids and metabolites. They are mediators of near and long-distance intercellular communication in health and disease, affecting several aspects of cell biology. This, together with their intrinsic features such as stability, biocompatibility, low immunogenicity and ability to overcome biological barriers, has prompted interest in using exosomes as drug delivery vehicles.

The most broadly studied EVs are exosomes, 40–150 nm in diameter, endosome-derived and originating from intraluminal vesicles (ILVs) that reside inside multivesicular bodies (MVBs) [18]. Nucleic acids and lipids are thought to be selectively and actively incorporated into ILVs [36]. Apart from the presence of membrane proteins, it is considered that inward budding of endosomal membranes integrates cytosolic proteins and other components to ILVs, after which MVBs fuse with plasma membrane, releasing ILVs as exosomes [18,37,38] (Figure 1A). Microparticles (MPs), microvesicles, also called ectosomes, are large EVs (100–1000 nm) shed directly by blebbing and budding mechanisms from the plasma membrane [39]. Apoptotic bodies are usually much larger (1000–5000 nm), composed of cellular and nuclear fragments, formed during the late stages of apoptosis [18,37]. Several molecular markers are rich in EV subtypes, such as alix, syntenin-1, TSG101 and CD81 for exosomes and metalloproteinases, integrins and phosphatidylserine for MVs [40,41].

When released to the extracellular space, exosomes interact with their acceptor cells in different ways. Information transmission can occur at the cell surface without delivering any cargo (surface binding), as occurs during immune responses [20,42], but the most common way is internalization of exosomes or their content, through clathrin, caveolin or lipid rafts-mediated endocytosis, phagocytosis, macropinocytosis or direct membrane fusion [43,44,45,46,47,48] (Figure 1A). The function of EVs in recipient cells depends on their cargo and thus on the cell type from which they are released. Cargos transported by EVs are highly heterogeneous and include RNAs (coding and non-coding), DNAs, lipids and proteins that can be transported inside EVs or membrane-bound [35,38]. RNAs enclosed in EVs such as mRNAs can control cell differentiation processes, survival, repair and angiogenesis [49,50,51,52] (Figure 1B). MiRNA are the most studied RNAs present in exosomes, which exert their function through gene expression regulation in acceptor cells [36]. EVs-miRNAs have revealed a role in a wide range of cell processes, both beneficial and detrimental, such as immune response, angiogenesis, apoptosis and differentiation [53,54,55]. Lipids transported by EVs are asymmetrically distributed between the outer and inner EV membranes and include cholesterol, ceramides, prostaglandins, sphingolipids and phosphatidylserines. Their functions have been related to the EV biogenesis itself and more importantly, to an immunomodulatory role of bioactive lipids EV-transported in immune-related pathologies [56]. The protein content of EVs is highly enriched in cytoskeletal, cytosolic, heat shock and vesicular trafficking proteins that control different cell signaling pathways including calcium signaling, coagulation and inflammation [57,58,59]. Therefore, EVs and particularly exosomes modulate a variety of physiological processes related to cell homeostasis and regulation, also mediating detrimental processes during disease.

Indeed, due to their involvement in normal and pathological cellular physiology, EVs have been recognized as good biomarkers in a wide variety of diseases including cancer, cardiovascular diseases, nephropathies and autoimmune diseases [60,61,62,63,64]. Moreover, exosomes have been considered useful indicators of disease progression. In cancer, tumor-derived exosomes are pointed out as biomarkers of disease development through tumor progression monitoring [65,66]. In autoimmune diseases, recent works show exosome-associated miRNA profiles as a promising tool for disease monitoring [29,30,67] and a prognostic tool for therapy response [68].

## 3. Role of Extracellular Vesicles in Systemic Lupus Erythematosus

### 3.1. Modulation of Immune Response

Systemic lupus erythematosus is a prototypic autoimmune disease characterized by diverse immune disturbances. One of the serologic hallmarks of SLE is the production of antibodies to nuclear molecules (antinuclear antibodies (ANA)) [3,4]. These ANA can form circulating proinflammatory immune complexes (ICs) that trigger cytokine production by innate immune cells or deposition in the tissue (especially the kidney) to fix complement and incite inflammation [69]; EVs could be mediators in these processes.

Several reports indicate higher levels of circulating IC-carrying MPs in SLE; as an example, Ullal et al. demonstrated that MPs display DNA and nucleosomal molecules in an antigenic form and could represent a source of ICs in SLE [70]. Another study by Nielsen et al. demonstrated that plasma MPs carry antigens accessible to autoantibodies and that complement-activating ICs may form on MPs in SLE patients [71]. Additionally, Cloutier et al. showed that platelet-derived MPs from synovial fluid carried large quantities of ICs, composed primarily of anti-citrullinated protein antibodies (ACPAs) directed against vimentin and fibrinogen (well established autoantigens in rheumatoid arthritis) and not by MP Fc-receptor binding [72].

Given their composition and immune properties, EVs act as proinflammatory mediators in SLE [73]. In one study, healthy peripheral blood mononuclear cells (PBMCs) were stimulated with exosomes isolated from SLE patients, producing TNF-α, IL-1β, and IL-6. Investigators demonstrated that circulating exosomes are immunologically active and their levels correlate with disease activity in lupus [26]. Dieker et al. reported that circulating apoptotic MPs from SLE patients drive the activation of dendritic cell subsets and prime neutrophils [74]. In another study, Winber et al. demonstrated that SLE patients display increased ROS production and degranulation by polymorphonuclear leukocytes (PMNs) in response to MPs [75]. Recently, Burbano et al. highlighted the involvement of platelet-derived MPs during monocyte activation in patients with SLE, showing that MPs are one of the most representative sources of the total amount of circulating ICs-IgG^+^ in patients with SLE [76]. Another study revealed that macrovascular and microvascular endothelial cells exposed to MPs and MPs-ICs from patients with SLE increase expression of adhesion molecules, chemokine production and structural alterations [77]. Finally, Salvi et al. reported that exosomes isolated from the plasma of SLE patients can activate secretion of IFN-a by human blood plasmacytoid dendritic cells (pDCs) in vitro. They identified exosome-delivered miRNAs as potentially novel TLR7 endogenous ligands able to induce pDC activation in SLE patients [27]. These findings enhance EVs as novel mediators during onset of autoimmune reactions and potential therapeutic targets in the SLE treatment.

### 3.2. Biomarkers and Predictors of Disease Activity

Several studies reported EVs as reliable biomarkers of disease activity beyond their role in regulating immune responses, offering a valuable complement to classical laboratory markers [64,78]. Nielsen et al. demonstrated different concentrations and composition of MPs in SLE patients from those in healthy subjects [79]. Additionally, a deeper phenotypic analysis of MP from SLE showed that MP were more abundant in these patients and were associated with declining renal function [80]. A proteomic study revealed a specific SLE-MP with a particularly altered proteome, including diminished mitochondrial and platelet proteins and increased glycolytic and cytoskeletal proteins [81]. Investigators concluded that an abnormal generation of MPs may partake in the pathology of SLE and that new diagnostic, monitoring and treatment strategies targeting these processes may be advantageous. In a recent study, MPs isolated from platelet-poor plasma and urine from SLE patients were characterized by flow cytometry, the authors concluding that HMGB1 + MPs present in urine are hallmarks of nephritis in patients with SLE [82]. 

Many recent studies highlight exosomal-derived miRNAs levels to identify distinct miRNA to discriminate LN. Our group revealed increased urinary exosomal miRNAs levels in patients with SLE, discriminating LN [28], and in a recent study, we identified urinary exosomal miR-146a as a marker of albuminuria, activity changes and disease flares in LN [29]. Another study reported a unique miRNA expression profile of urinary exosome (miR-3135b, miR-654-5p and miR-146a-5p) as novel non-invasive diagnostic markers for type IV LN with cellular crescent [31]. In line with early renal fibrosis, Solé et al. identified urinary exosomal miR-29c as a novel non-invasive marker of early progression to fibrosis in patients with LN [83]. Moreover, a urinary exosomal multimarker panel composed of miR-21, miR-150 and miR-29c was provided as a non-invasive method to detect early renal fibrosis and predict disease progression in LN [30]. Finally, a recent study reported urinary exosomal miR-135b-5p, miR-107 and miR-31 as promising novel markers for clinical outcomes, regulating LN renal recovery by HIF1A inhibition [32]. 

In summary, quantity and phenotype of circulating EVs may be useful as novel biomarkers of activity and progression of SLE, providing a new therapeutic approach.

## 4. Key Points in Exosomes as Drug Delivery Vehicles 

Research interest in exosomes has grown dramatically during the last few years due to their unique properties. Transport inside exosomes allows concurrent intercellular communication by delivering different signals simultaneously. Unlikely free circulating soluble factors, they have the ability to release large amounts of functional molecules to recipient cells [84]. Moreover, cargos enclosed inside exosomes are protected against degradation by enzymes and other processes by their lipid bilayer, conferring them stability and safety. Additionally, exosome properties allow cargos to travel long distances, have good biocompatibility, are non-immunogenic, specific-targeted and cross many physical barriers [84,85,86] (Figure 1B). For these reasons, exosomes are safe and stable endogenous nanocarriers and one of the best drug delivery systems options, with an increasing variety of applications. 

**Figure 2:**
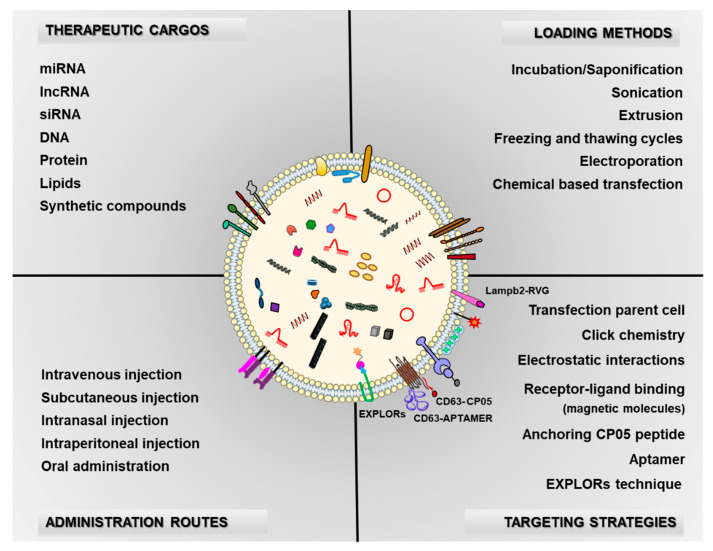
Schematic illustration of the key points in exosomes as drug delivery systems. Therapeutic cargos, methods for loading exosomes with these cargos, the use of targeting peptides on the exosome surface and routes of administration to reach the area of interest, are important issues that still need to be deeply addressed.

The potential use of exosomes in therapy is based on the cargos they deliver to recipient cells. Endogenous cargos with known beneficial properties or artificially modified molecules can be loaded using a range of methods. Likewise, different exosome administration routes for therapy purposes have been described, with novel methods being developed during recent years (Figure 2).

### 4.1. Therapeutic Cargos

Since of exosomes have an inherent ability to carry different types of active molecules, they can be biologically or chemically loaded to deliver enhanced or broadened therapeutic compounds, representing an informed way to treat many diseases. Research as drug delivery vehicles is based mainly on the transfer of small RNAs, although other possibilities are being explored.

#### 4.1.1. microRNA (miRNA)

Exosome based-miRNA therapy is the most developed area so far, since miRNAs function as gene expression regulators and have been implicated in the pathophysiology of numerous diseases. In addition, exosomal ability to carry therapeutic miRNAs has been extensively analyzed, demonstrating high effectiveness [87]. This therapeutic approach has been explored mainly for cancer therapies [88,89]. One of the first studies engineering exosomes with miRNAs for treatment purposes loaded miR-let7a inside GE11 peptide-positive cells, a modification designed to target epidermal growth factor receptor (EGFR)-positive cells, expressed in a high variety of tumors such as in breast cancer. The delivered miR-let7a lead to inhibition of tumor development. [90].

Similarly, mesenchymal stem cell (MSC)-based therapies have also demonstrated protective effects by promoting tissue repair, apoptosis and fibrosis inhibition or stimulation of cell proliferation [91,92]. MSCs are a rich source for exosome production [93], and their beneficial effects have been shown to be due in part to EV secretion [94]. In the last few years, growing interest on MSC exosomal cargo characterization has brought insight into mechanisms of action and development of directed therapies, most of them based on miRNAs [95,96]. As an example, Chen et al. showed that bone marrow MSC-secreted exosomes carrying miR-125b were able to protect against myocardial ischemia reperfusion injury by targeting SIRT7 [97].

Preliminary studies of the time laid the groundwork for pursuing exosomes as the best drug delivery system option for miRNA therapy, and paved the way to develop multiple strategies for refinement of methods and for direct targeting of exosomes to cell types.

#### 4.1.2. Small Interfering RNA (siRNA)

Small RNA interference (siRNA) has become a promising therapeutic strategy for knocking down targeted genes; however, their use as therapeutic compounds has limitations, as they are extremely hydrophilic and easily degraded [98]. Moreover, artificial methods to deliver siRNAs have shown to be toxic for most cell types and reach low transfection efficiency rates [99]. The use of exosome-enriched siRNAs offers the possibility to safely transport and deliver the interfering RNA specifically to a desired recipient cell, where circulating siRNAs would be rapidly degraded. Evidence of efficient delivery of siRNAs from exosomes and protocols to specific target cell types has previously been reported [100,101]. The exact mechanisms for siRNA packaging into EVs are not yet elucidated; however, Reshke et al. have developed a strategy for siRNA production, reaching high silencing at low doses [102]. Many studies describe the therapeutic use of siRNA-exosomes, especially in cancer. Recently, Xu et al. performed a pancreatic cancer siRNA-based therapy targeted to the PAK4 oncogene. They packaged the PAK4-siRNA in exosomes for delivery to tumor cells in vitro and in vivo and showed reduced tumor growth and enhanced survival in mice [103]. Similarly, Kamerkar et al. engineered exosomes with KRAS-siRNA against a specific oncogenic mutation and showed cancer suppression and enhanced survival [104]. Apart from the promising results obtained for therapy in cancer, these studies conducted in-depth analysis of the effectiveness of exosomes and showed a high efficiency compared to traditional transfection methods [102,103,104]. Despite the encouraging efficacy of this approach, the therapeutic potential of siRNA-exosomal delivery in diseases apart from cancer remains understudied [105,106]. In one example, exosomal siRNA-HMGB1 with a modified surface to study delivery efficiency for therapy in ischemic brain stroke. Compared with unmodified exosomes, the modification induced greater reduction of apoptosis and infarct size in the murine model [107]. These modified properties on exosomes are currently being explored in different approaches, and as outlined below open a broad range of possibilities.

#### 4.1.3. Long Non-Coding RNAs (lncRNAs)

There is emerging interest in long non-coding RNAs not only as biomarkers but also as therapeutic agents due to their ability to interact with miRNAs. lncRNAs are naturally carried by EVs and are been increasingly documented as key mediators of EV biological effects [108]. Therapeutic applications of lncRNAs enclosed inside EVs span a broad range including cardiovascular, musculoskeletal, gastrointestinal and nervous system diseases, in which they have demonstrated potential use in mediating tissue regeneration and repair [109,110]. Applications in cancer therapy have also been developed [111,112]. Curiously, exosomal lncRNA-H19 has also been reported as a potential therapy in different diseases. In a myocardial infarction rat model, MSC-derived exosomes pre-treated with atorvastatin showed cardioprotective effects by inducing increased levels of lncRNA-H19, involved in miR-675 regulation [113]. In diabetes, lncRNA-H19 engineered in EV-mimetic nanovesicles demonstrated a neutralizing power in the regeneration-inhibiting effect of hyperglycemia, and accelerated the healing processes of chronic wounds [114]. Finally, lncRNA transported inside EVs from human adipose-derived stem cells showed increased survival rates in an acute liver failure rat model [115]. 

#### 4.1.4. Different DNA Species 

As shown before, exosomes are promising targets for therapeutic delivery based on small nucleic acids such as siRNAs and miRNAs, which are successfully loaded inside EVs. However, the use of exosomes as drug delivery systems of exogenous DNAs remains poorly researched despite the widely reported presence of different DNA species inside exosomes [116,117]. Loading efficiency depends on both DNA and EV size, so that larger DNA molecules (more than 1000bp) or plasmids are much more difficult to load and the use of smaller EVs is limited to smaller sized DNA. Moreover, the first studies conducted in this field showed efficient exogenous DNA transfer by different methods, but functional gene transfer was not observed in all cases [118,119]. Despite this, novel techniques have been developed and there are an increasing number of studies based on exosome-DNA transfer for therapy [120,121]. In this light, Kanada et al. engineered minicircle DNA inside EVs encoding prodrug converting enzymes for mammary cancer therapy which resulted in tumor cell death [122]. Although in-depth research in needed on this approach to develop more efficient techniques, the use of EVs for DNA delivery represents a promising alternative to adenoviral vectors, which have major limitations, partly due to their toxicity and immunogenicity.

#### 4.1.5. Proteins

In addition to different nucleic acids, exosome transported proteins are also being used as therapeutic cargos, albeit less commonly [123,124]. Aspe et al. showed that the exosome-mediated delivery of survivin-T34A to pancreatic cell lines produced an increase in gemcitabine sensitivity, improving the efficacy of chemotherapy-induced apoptosis [125]. Recently, Yang et al. have demonstrated that simultaneous delivery of nerve growth factor mRNA and protein in ischemic regions of cerebral stroke mice ameliorated inflammation and reduced cell death [50].

#### 4.1.6. Synthetic Therapeutic Compounds

Apart from the natural cargos of exosomes (miRNAs, siRNAs, DNAs, proteins, etc.), their excellent biological properties have driven their use as drug delivery systems of synthetic compounds (see [126] for review). The carriage of chemical drugs by exosomes is being exploited mainly in cancer, using chemotherapy agents as cargos. For instance, doxorubicin-encapsulated exosomes showed efficient targeting to HER2+ and αv integrin-positive breast cancer cells in vitro, and in an in vivo mice model, injected targeted exosomes delivered doxorubicin specifically to tumor tissues inhibiting tumor growth [127]. Moreover, a recent study from Zhan et al. reported a novel combination of chemotherapy with gene therapy with the co-delivery of doxorubicin and miR-21 inhibitor [89]. Phytochemical therapeutic cargos such as paclitaxel, celastrol and curcumin have been successfully loaded into exosomes and exercised a potent anti-cancer effect with enhanced anti-inflammatory power and response to tumors [128,129,130]. The ability of exosomes to effectively cross the blood–brain barrier have singled them out as potential drug nanocarriers for brain inflammatory or neurodegenerative diseases. This is the case for exosome-loaded dopamine in treatment of Parkinson’s disease, which showed a both in vitro and in vivo 15-fold increase in delivery efficiency to the brain, mediated by exosomes [131]. Less studied, but with encouraging results, is the use of exosome-encapsulated synthetic compounds for regeneration therapies, to transport titanium nanotubes implants inside exosomes for bone regeneration [132]. 

### 4.2. Methods for Loading Exosomes with Therapeutic Cargos 

Several methodological approaches (exogenous or endogenous) have been attempted to improve the efficiency and specificity of EVs delivery. Exogenous loading after EV isolation, which can be divided into passive and active processes, includes incubation as a passive option and sonication, extrusion, freezing and thawing cycles, electroporation and chemical-based transfection as active processes where the use of electric field or surfactants are required for EV membrane permeabilization. Endogenous manipulation refers to introducing the cargo of interest into EV-producing cells, commonly transfecting cells with expression vectors. In addition, modification or attachment of molecules and receptors to EV surface are key ways to enhance delivery of targeted therapy [133].

#### 4.2.1. Incubation

Passive loading of EVs can be simply performed by incubating exosomes with the cargo of interest. The differing concentration inside and outside the EVs will drive diffusion of hydrophobic cargos through EVs lipid-bilayer. Several chemotherapeutic drugs such as doxorubicin and paclitaxel have been loaded into exosomes by incubation [127,134], demonstrating enhanced chemotherapeutic effects [135]. Different cargos such as enzymes have also been loaded into exosomes using this method for Parkinson’s disease therapy [123]. Additionally, Qu et al. improved dopamine loading efficiency by incubating it in a saturated solution [131]. This method is simple, cost-effective and compatible with hydrophilic molecules, but unable to load hydrophobic ones.

Saponification allowed up to 11-fold higher drug loading of large or hydrophilic compounds like porphyrins compared to passive methods [136]. In this sense, incubation with surfactants such as saponin, which interacts with cholesterol increasing membrane permeability, resulted in highest loading efficiency without alterations in exosomes size or morphology [123]. However, detergent elimination is necessary to assure exosome integrity. 

#### 4.2.2. Sonication 

An additional loading technique is to apply sound energy with a sonicator to exosomes solution, which induces mechanical shear forces affecting exosome membrane integrity and enhancing the incorporation of the cargos present in the solution to vesicles [137]. This process does not report alterations in exosomes integrity or native cargos, but incubation at room temperature after sonication is recommended for membrane recovery [134]. Sonication resulted in more efficient loading compared with incubation with or without saponin [123], but could cause unspecific release of the drugs attached to the outer exosomal membrane. 

#### 4.2.3. Extrusion

Extrusion is a process by which exosomes are loaded by membrane disruption when extruded through small polycarbonate porous membranes, allowing to incorporate cargos present in the solution to the vesicles [123]. Despite the use of this technique is not very popular, a recent study suggests its applications such as interesting and promising method for exosomes loading without alter exosomes characteristics [138].

#### 4.2.4. Freeze-Thawing Cycles

This method consists of freezing and subsequently thawing a solution of vesicles with the cargo of interest. The mix is incubated at 37 °C, frozen rapidly at −80 °C and then thawed at room temperature, repeating at least three times. One inconvenience of this method, however, is aggregation of vesicles into large-sized particles [123,138].

#### 4.2.5. Electroporation

Permeabilization of EV membrane by applying an electrical field is one of the most common techniques employed to enhance uptake of the cargo of choice. Disruption of the phospholipid bilayer of EVs allows the hydrophilic compounds to be diffused into exosomes such as small DNAs [119,139], miRNAs [140,141] and siRNAs [107,142]. Nevertheless, although electroporation can improve loading efficiency as well as passive incubation, this technique has various disadvantages, principally aggregation of siRNAs and vesicles themselves [143,144].

#### 4.2.6. Chemical-Based Transfection

Use of chemical reagents for siRNA loading into exosomes has also been described. Wahlgren et al. employed liposome-based transfection reagent to introduce MAPK-1 siRNA into exosomes by incubating the mixture for 10 min at room temperature [145]. Likewise, Shtam et al. transfected two different siRNAs against RAD51 and RAD52 into exosomes by liposomes, resulting in effective post-transcriptional gene silencing in the recipient cells [100]. Although these reagents are popularly used for high success transfection in in vitro experiments, their efficiency is worse than electroporation. In addition, their toxicity, low exosomes loading capacity and the difficulty of distinguishing between exosomes and derivate reagent aggregations makes this approach unsuitable for therapeutic purposes.

#### 4.2.7. Engineering Exosome-Producing Cells

Another interesting approach is the generation of engineering cells to produce loaded exosomes with specific cargos. One approach is transfection of donor cells to overexpress a particular gene, allowing the generation of specific gene-loaded exosomes during their biogenesis. Jiang et al. investigate the therapeutic effect of TSG-6 modified MSC-derived exosomes on a mouse full-thickness wound model, providing evidence of a possible mechanism to prevent from scar formation [146]. In addition, several studies showed that MSC-secreted exosomes carrying miRNAs represent a new strategy to improve treatments [147]. In a recent article, Lou et al. determine that exosomes from adipose tissue-derived MSCs can be used to deliver miR-199a and improve HCC chemosensitivity by targeting mTOR pathway [148]. In another study, Chen et al. showed that bone marrow MSC-secreted exosomes carrying miRNA-125b are able to protect against myocardial ischemia reperfusion injury via targeting SIRT7 [97]. Finally, therapeutic molecules can be loaded in MVs following an endogenous approach, drugs are first loaded into parent cells, followed by the generation of drug-loaded MVs. This approach depends on the cellular machinery allowing the encapsulation of external compounds. MSCs incubated with paclitaxel release paclitaxel-loaded MVs that exert cytotoxicity to tumor growth in vitro [149]. Upon ultraviolet light irradiation, tumor cells loaded with chemotherapeutic agents release MVs that are functionally active against tumors [150].

### 4.3. Different Exosome Administration Routes to Reach the Disease Area 

In addition to isolating, loading and/or modifying exosomes, selecting the exosome administration route and dose are also important parameters to consider to achieve clinical relevance in the future. In this respect, a key challenge is enhancing the biodistribution, stability and therapeutic effect of exosomes. In the following subsection we summarize some of the most frequently used exosome administration routes. 

#### 4.3.1. Intravenous Injection

Systemic injection is one of the most commonly administration routes used in exosome therapeutics [90,106]. Amelioration of myocardial injury derived from infarction [151], neuroprotection against stroke injury [152] and reduction of glioblastoma size tumors [153] are examples of therapeutic approaches developed via intravenous injection. However, intravenous administration results in accumulated exosomes in several organs, preferably in liver and lungs for their metabolic clearance action, which eliminates vesicles rapidly from blood. In spite of their prompt clearance from circulation, methodological alternatives such as exosome surface modification with polyethylene glycol (PEG) have shown to enhance the half-life of vesicles in circulation from 10 to 60 min [154]. In the context of autoimmune diseases, Wen et al. elucidated a mechanism by which therapy with bone marrow MSCs and PBMC co-cultured exosomes with suppressed immune reaction after islet transplantation, inhibiting PBMC proliferation and improving T-cell regulatory functions [155]. Furthermore, Riazifar et al. found that systemic injection of IFNγ-Exo resulted in sustained clinical recovery with enhanced motor skills, a decrease in neuroinflammation and reduced demyelination in an autoimmune encephalomyelitis mouse model [156].

#### 4.3.2. Subcutaneous Injection

Several studies provide evidence that exosomal subcutaneous injections are effective in several pathologies [157,158], but especially in cutaneous malignancies. Recent studies have described the therapeutic potential of exosomes to enhance wound healing after cutaneous treatment of injured areas. As an example, subcutaneous administration of M2 macrophages-exosomes into the wound edge, contributed successfully to an exosome-guided switch to M2 Mϕ polarization, which accelerates wound healing by enhancing angiogenesis, re-epithelialization and collagen deposition [159]. In addition, presence of miR-221-3p in endothelial progenitor-derived exosomes, promoted skin wound healing when administrated in scars of normal and diabetic mice [160].

#### 4.3.3. Intranasal Injection

Intranasal injection provides a practical option for administration of therapeutic compounds to the brain. One of the first studies reported that curcumin-loaded EL-4-derived exosomes were efficiently delivered to microglia cells by intranasal administration, showing protection against lipopolysaccharide (LPS)-induced brain injury [161]. In addition, intranasal injection of exosomes has shown to prevent neurodegeneration [162]. In more detail, catalase-loaded exosomes in a Parkinson’s disease mouse model showed an increase in the quantity of exosomes with catalase-preserved activity detected in the brain and generated more neuroprotective effects than when administrated intravenously [123]. Recently, Ezquer et al. observed that intranasal injected MSC-derived exosomes reduced oxidative stress and inhibited ethanol consumption in an animal model of chronic alcohol consumption [163]. 

#### 4.3.4. Intraperitoneal Injection

Although not commonly used as intravenous injection, intraperitoneal delivery of exosomes is another administration route option [130]. Interesting results were obtained in several studies after intraperitoneal injection, such as recognition of chronic myeloid leukemia cells and inhibition of cancer proliferation [164], and prevention of bronchopulmonary dysplasia [165]. Furthermore, Nojehdehi et al. demonstrated that intraperitoneal injection of adipose MSC-derived exosomes results in an immunomodulatory effect on autoimmune type 1 diabetes, increasing regulatory T-cell population and their products without a change in lymphocyte proliferation [166].

#### 4.3.5. Oral Administration 

In addition to the administration routes previously described, oral treatment offers a simple non-invasive route of exosome delivery. This method, like others summarized above, has been employed to administrate chemotherapeutic drugs such as curcumin, resulting in three- to five-fold higher levels of curcumin in several organs compared to free delivery of the compound [167]. Regarding autoimmune diseases, Arntz et al. described for the first time that oral delivery of bovine milk exosomes delayed the onset of rheumatoid arthritis (RA), showing diminished cartilage pathology and bone marrow inflammation, together with a reduction in MCP-1 and IL-6 serum levels [168]. Recently, an immune tolerance mechanism mediated by free light chain-coated, antigen-specific, miR-150-carrying exosomes that act on the antigen-presenting cells proved very effective after oral administration [169].

From our literature review, we found a paucity of data comparing exosome loading by these different methods. Thus, it will be a vibrant area of research to assess the optimum doses required in vivo. In addition, the efficacy and safety of cargo-exosome delivery should be characterized in association with other available methods for gene and drug delivery to discover significant breakthroughs as well as find innovative applications of this novel approach.

## 5. Modified Exosomes for Drug Delivery

The properties of exosomes alone are not sufficient to guarantee the specific delivery of drugs and the enrichment of drugs in disease tissues. Indeed, a wealth of literature supports that exosomes need targeting strategies to improve the therapeutic effect of drugs (Figure 2).

Targeted delivery of exosomes with increased biodistribution and specificity can be assessed by transfection of parent cells. The fragment is transfected by fusing the ligand of interest to the coding sequence of exosomal signaling peptide, allowing the protein of interest to attach to the vesicle surface [170]. The most frequently used exosomal transmembrane proteins include tetraspanins [171], lysosome-associated membrane protein 2b (Lamp2b) [106,107,172], glycosyl-phosphatidyl-inositol [154], platelet-derived growth-factor receptors [90] and lactadherin [173]. In a pioneering study, Alvarez–Erviti et al. fused rabies viral glycoprotein (RVG) with Lamp2b to deliver exosomes specifically to neurons and glia. Targeted exosomes successfully accumulated in the target tissues and improved cargo delivery [106]. After this study, this methodology was further employed to fuse different proteins to Lamp2b such as αv integrin-specific iRGD peptide [127], and cardiac targeting peptide [174], all with promising results.

Direct functionalization of exosomes is another option for exosome surface modification. Functionalization strategies tested, include covalent and non-covalent chemical. Covalent modification includes copper-catalyzed azide-alkyne cycloaddition (CCAAC) click chemistry, the reaction of an alkyne and an azide chemical group to form triazole linkage [175]. The process presents several advantages over the gene editing of parent cells: Faster reaction times, high specificity and compatibility with organic and aqueous buffers [176,177,178]. Metabolic labeling of parent cells is a second strategy inside covalent surface modification methods. Supplementation of cell culture medium with synthetically modified amino acids, lipids, glycans or oligonucleotides, leads to the incorporation of these compounds on the cell metabolism and consequently, on the surface of produced exosomes. Moreover, the presence of these selected domains into vesicle surface allowed to employ the click chemistry method described to bind fluorescent dyes to exosomes [179].

Along with the surface modifications described, stable modification of the exosome surface by non-covalent alteration of the EV membrane has been also developed and is summarized below. 

Multivalent electrostatic interactions involve binding highly cationic species to negatively charged groups located in the exosome membrane [180]. However, the major downside to this approach is cytotoxicity of cationic nanomaterials caused by membrane thinning and hole formation. A second non-covalent strategy involves receptor–ligand binding, where natural receptors present over the exosome membrane are used to attach targeting ligands. Zhan et al. developed a new gen/chemo combination therapy whereby doxorubicin and cholesterol-modified miR-21 inhibitor were co-embedded into the lipid bilayer of exosome. In addition, magnetic molecules and endosomolytic peptides L17E were bounded to the exosome membrane through ligand–receptor coupling and electrostatic interactions [89,181]. Nonetheless, this strategy presents certain drawbacks such as the synthetic challenge and cost of presenting functional ligands (e.g., transferrin, biotin) on the exogenous material. Fusion of lipid-based particles such as liposomes or micelles with exosomes through hydrophobic interactions is another non-covalent surface modification approach. Previous functionalization of the liposomal membrane followed by its fusion with exosomes have demonstrated to be effective for the integration of lipophilic species into exosomes without affecting their native functions or integrity [182]. Additionally, aptamer-based modification represents in situ assembly method based on molecular recognition between DNA aptamers and exosome surface markers such as CD63, shedding light on new useful methodologies for exosomes targeting [183]. Finally, modification by anchoring CP05 peptide has been demonstrated effective at enhancing targeting, loading and purification of exosomes by binding to CD63 exosome marker [184]. Interestingly, Yim et al. described a revolutionary optogenetic exosome system, exosomes for protein loading via optically reversible protein–protein interaction (EXPLORs) [185]. By integrating a reversible protein–protein interaction module controlled by blue light with the endogenous process of exosome biogenesis, cargo proteins were successfully loaded into newly generated exosomes.

Development of modified exosomes has an auspicious future in drug delivery research. Innovative targeting moieties should be designed to promote enhanced delivery to a specific cell type. Although researchers have made impressive progress in the modification of engineered exosomes, most of the above-mentioned techniques are based on the modification of exosome donor cells. These changes may affect the protein composition and function of exosomes. Therefore, it is necessary to explore new techniques for exosome-targeted modifications to minimize changes in their composition. Maintaining progress in these areas will bring breakthroughs in the field and transform exosomes from promising candidates into smart nanoscale therapeutics.

## 6. Exosome-Based Drug Delivery in Systemic Lupus Erythematosus

Both in themselves and as vehicles of drug and gene delivery, exosomes are under active development as therapeutic agents [17,186]. Exosomes loaded with endogenous and/or exogenous cargo have recently emerged as novel therapeutic effectors in immune therapy [24,187], targeting the autoimmune-mediated inflammatory pathology associated with SLE [188]. Exosomes can modify major components of innate and adaptive immune responses, including T-cells, B-cells and macrophages [189,190,191]. This immunomodulatory property makes exosomes an attractive tool for immunotherapy as well as tissue regeneration.

The use of EVs as a cell-free therapeutic alternative offers several distinct advantages over parent stem cells. A major advantage is that EVs, depending on their source, may be less immunogenic than their parental cells, likely due to a lower abundance of transmembrane proteins such as MHC complexes [192]. Unlike live cells, EVs are highly stable and easily stored long-term. In addition, EVs do not replicate, thus avoiding risk of aneuploidy or other chromosomal abnormalities in tumor generation. Finally, exosomes are also able to cross biological barriers that MSCs cannot pass [85,106], a significant advantage in a systemic disease such as SLE which affect organs with physiological barriers as the brain and kidney (blood–brain and blood–urine). 

**Figure 3:**
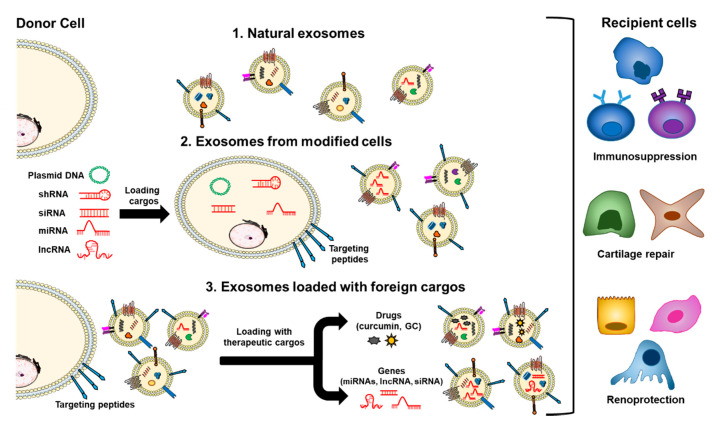
Natural exosomes and engineered exosomes loaded with endogenous or exogenous cargos for therapeutic purposes. Immune-therapeutic exosomes include naturally occurring exosomes mainly from mesenchymal stem cells (route 1); exosomes secreted by modified cells due to pathological factors or transfection (route 2); and exosomes loaded with exogenous cargos classified into three kinds, mainly including small molecule drugs, such as curcumin and glucocorticoids (GC), nucleic acids (miRNA, small interference RNA (siRNA), lncRNA, etc.), proteins and peptides (route 3). These exosomes can be surface-modified with targeting peptides for a successful exosome-based drug delivery system. In the context of systemic lupus erythematosus, immunosuppression, cartilage repair and renoprotection are important pathways regulated in target cells by exosome-based drug delivery. shRNA: Small hairpin RNA.

Immune-therapeutic exosomes include naturally occurring exosomes, exosomes secreted by modified cells, and exosomes loaded with exogenous cargos (Figure 3).

### 6.1. Therapeutic Application of Naturally Secreted Exosomes 

Several studies have shown that EVs from various cell sources have a therapeutic effect through their intrinsic content [193], which includes tumor-derived EVs, MSCs [93], activated antigen-presenting cells (APCs), natural killer (NK) cells and endothelial progenitor cells (EPCs) (Figure 3, route 1). Furthermore, cell-derived exosomes can improve immunosuppressive ability after cell pretreatment. With the stimulation by physical and chemical factors, exosomes can produce stronger immunosuppressive effects [191]. In autoimmune diseases, including SLE, the most studied naturally secreted EVs for therapeutic purposes derive from MSCs and EPCs.

One major role of MSCs is to suppress proliferation and function of cells in both innate and adaptive immunity responses [194]. Thanks to this characteristic, they are extensively studied for their therapeutic advantages in a variety of inflammatory and autoimmune diseases, including SLE [188]. Previous data from SLE animal models showed a significant decrease in autoantibody production, proteinuria and glomerulonephritis [195,196], and B-cell activation was also suppressed [197]. In addition, a number of molecules released by MSC are packaged into EVs, so MSC-derived EVs (MSC-EVs) possess similar immunomodulatory properties to MSCs [198,199]. 

MSC-EVs can exert immunosuppressive effects on T cells [198], inhibiting activation and development of T cells by interferon-γ [200]; on B cells [201], reducing production of immunoglobulin and inhibiting their proliferation and differentiation [202]; on macrophages, inducing a phenotypic transition of macrophages from M1 to M2 dendritic cells (DCs) [203]; and on DC [22,204], and on NK cells [205]. Furthermore, several reports highlight the efficacy of human MSC-exosomes in reducing kidney inflammation and maintaining joint integrity, which are both target areas of SLE pathology [206,207,208,209]. Tomasoni et al. demonstrated that MSC-EVs provide powerful renoprotection via horizontal transfer of the mRNA for IGF-1R to tubular cells [210]. In another study, Shen et al. demonstrated that receptor proteins of MSC-exosomes such as C-C motif chemokine receptor-2 (CCR2) suppress macrophage functions and alleviate renal injury [211]. Finally, EPC-EVs prevent renal tissue injury by delivering their RNA content, the miRNA cargo [212]. In a rat model of experimental glomerulonephritis, EPC-EV-carried RNAs inhibited leukocyte infiltration, mesangial activation, activated serum complement and decreased proteinuria, improving renal function [213].

### 6.2. Exosomes Secreted by Modified Cells

Loading therapeutic cargos into exosomes is based on their biological processes, cargo is loaded into donor cells or overexpressed certain gene products which are then packaged into exosomes (Figure 3, route 2). Exosome content of cells modified by pathological factors (physical/chemical factors) or transfection can be delivered to recipient cells as a therapeutic method [191].

MSC is the most well-suited for mass production of exosomes for drug delivery [93]. For example, Tavasolian et al. showed that manipulation of MSC-derived exosomes with anti-inflammatory miR-146a increases Treg cell populations and anti-inflammatory cytokines, promoting the recovery of appropriate T-cell responses in inflammatory situations such as RA or SLE [214]. Several studies investigated the therapeutic potential of MSC-derived miRNAs expressing exosomes in RA. MiR-150-5p reduced joint destruction by inhibiting synoviocyte hyperplasia and angiogenesis, targeting MMP14 and VEGF [215], and miR-192-5p delayed the event of the inflammatory response, targeting ras-related C3 botulinum toxin substrate 2 (RAC2) [216]. In another study, Meng et al. reported that MSC-derived exosomes loaded with miR-320a participate in the intercellular transfer of miR-320a and subsequently inhibit the progression of RA by suppressing CXCL9 expression [217]. In kidney disease associated with inflammatory processes, a previous work from Wang et al. showed that MSCs, engineered to overexpress miR-let7c, selectively homed to damaged kidneys and attenuated kidney injury through an effective anti-fibrosis function [218]. Zhu et al. demonstrated that exo-MSCs protect against renal ischemia/reperfusion injury by transferring miR-199a-3p which reduces apoptosis in renal cells, downregulating Sema3A expression and thereby activating the AKT and ERK pathways [219]. Finally, recent studies showed that miR-140-5p, miR-92a-3p and miR-210-overexpressing MSC protect against chondrocyte injury, suppressing cartilage degradation [220,221,222].

### 6.3. Exosome Loading with Exogenous Cargos (Drugs or Genes) for Therapy

Exogenous cargos can be directly loaded into exosomes by different methods (see Section 4.2), and the cargo can be classified into three kinds, mainly including small molecule drugs, nucleic acids (miRNA, siRNA, lncRNA, etc.), proteins and peptides. However, using the properties of exosomes alone is insufficient to achieve specific delivery of exogenous cargos into diseased tissues. Therefore, as described before, different exosome engineering techniques to improve the therapeutic effect of exosomes are still being developed, such as surface modification of exosomes or loading magnetic nanoparticles [17,84,181,223]. These targeting strategies for exosome carriers improve the delivery of specific therapeutic foreign substances in several diseases such as myocardial infarction [224], cerebral ischemia [225], Alzheimer’s disease [106] and multiple types of cancer [104,226]. Recent works have pinpointed exosomes loaded with foreign cargos for immunotherapy in autoimmune diseases.

Encouraging recent studies show exosomes as potential therapeutic vehicle systems for small molecule drugs to reduce toxicity and enhance target disease tissue in autoimmune diseases. Currently, GCs are the most effective anti-inflammatory drugs available for SLE [6], but clinical application is limited by their nonspecific distribution after systemic administration and serious adverse reactions during long-term administration. A recent study by Yan et al. provides a promising strategy using exosomes as nanocarriers to enhance the therapeutic effect of GCs against RA [227], establishing a biomimetic exosome encapsulating dexamethasone sodium phosphate nanoparticle, whose surface was modified with folic acid (FA)-PEG-cholesterol (Chol) compound.

Another potential drug to load in exosomes is curcumin, which has immunomodulatory potential in addition to anti-oxidant and anti-inflammatory effects. In both acute and chronic immune nephritis curcumin ameliorated kidney disease reducing proteinuria, glomerulonephritis, tubule-interstitial disease and renal infiltration [228]. In addition, curcumin attenuates LN by inhibiting macrophage activation and macrophage-secreted B-cell activating factor (BAFF) [229], and reduces angiotensin II-induced podocytes injury and apoptosis, inhibiting endoplasmic reticulum stress [230]. One of the main limiting factors in the clinical use of curcumin is its poor bioavailability and rapid elimination. In this context, Sun et al. have demonstrated that the anti-inflammatory activity of curcumin is enhanced when encapsulated in exosomes, resulting in protection against lipopolysaccharide-induced septic shock in mice [130]. The use of exosomal curcumin may take advantage of functional properties of curcumin and exosomes in an additive way to fight inflammation related SLE disease.

Delivering specific functional nucleic acid drugs to targeted cells by exosomes can regulate gene expression and maintain the physiological balance of the cells [95,231]. Liu et al. have recently reported that exosomal lncRNA-KLF3-AS1 derived from MSCs can promote chondrocyte proliferation via miR-206/GIT1 axis. Additionally, in vivo studies demonstrated that exosomal KLF3-AS1 promoted cartilage repair [232,233]. In another study, Liang et al. demonstrated that by fusing a chondrocyte-affinity peptide (CAP) with the Lamp2b on the surface of exosomes, CAP-exosomes efficiently encapsulated with miR-140, delivered the cargo into chondrocytes and inhibited cartilage degradation [234]. Likewise, a recent report showed that exosomes isolated from adipose derived-MSC transfected with miR-10a promoted Th17 and Tregs response while reducing Th1 response, indicating new therapeutic potential, particularly as regards novel immunotherapeutic strategies [95]. Delivery of nucleic acid drugs involves fundamental treatment at the genetic level of autoimmune diseases. However, the precise mechanisms, method s of use and effects as regards safety considerations still require more in-depth exploration.

Exosomes also transported protein cargos, including shock proteins, major histocompatibility complexes, cytoskeletal proteins and proteins related to signal communication and membrane transport [235,236]. In this context, recent studies have demonstrated that exosomes with engineered surface or loaded with exogenous proteins play a role in apoptosis and tumor cell antigen presentation [125,237,238]. Peptide cargos, are generally found in antigen-antibody interactions and have an important role in the cellular immune response. Research on exosomes loaded with peptides or protein is making headway and this line of study should be explored in the context of autoimmune diseases such as SLE.

In summary, these works illustrate the undeniable potential of exosomes as a therapeutic drug carrier in autoimmune diseases (Table 1). Nevertheless, more studies are warranted for a clear understanding of the application of exosome-based drug delivery in autoimmune diseases, overall, in SLE.

## 7. Conclusions and Future Perspectives

Most autoimmune disorders such as SLE are characterized by a chronic inflammatory state, the making reduction of inflammation essential in order to treat patient conditions. Drug therapies for controlling SLE have made significant progress in recent decades. However, dosing limits of therapeutic indications in current SLE therapeutics delay their optimal use, and SLE patients may tolerate severe negative side effects produced by non-specific organ toxicity from frequent and long-term treatment. Compared with conventional therapeutic approaches, exosome-based drug delivery system has many advantages, such as high stability, compatibility, low immunogenicity and toxicity, longer circulation time and targeting ability for specific drug delivery to inflamed tissue. Therefore, exosome-based nanocarriers potentially have a bright future as next-generation drug delivery vehicles. This review has provided the current state of the art in the field of exosome-based drug delivery in SLE, evaluating the beneficial effects of natural and engineered exosomes on immunosuppression, cartilage repair and renoprotection.

**Figure 4:**
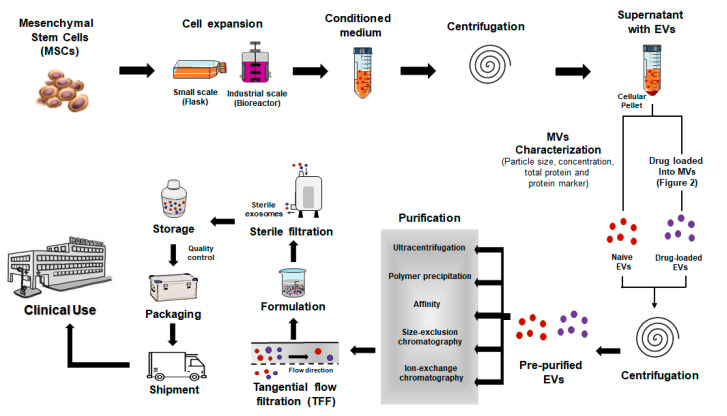
Clinical manufacturing approaches to produce exosome-based therapeutics. Large-scale good manufacturing practice (GMP)-exosome production under GMP-compliant procedures to ensure the quality, safety and consistency. EVs: Extracellular vesicles.

However, the clinical manufacturing of exosome-based therapeutics requires that comply with good manufacturing practice (GMP) to ensure reproducibility, stability and purity (Figure 4). Therefore, several shortcomings and obstacles need to be overcome to bring the maximum potential of the exosome-based drug delivery system to the clinic: (i) precise understanding of exosome biogenesis and targeting; (ii) a standardized isolation and purification protocol for reproducibility of exosomes; (iii) commercial production of exosomes for large-scale manufacturing; (iv) evaluation of the effects and mechanisms underlying these effects in vitro and in vivo, then in human diseases; (v) innovative approaches for exosome targeting strategies to optimize exosome loading efficiency, evaluating the stability of the loaded agent and modifying exosome properties; and (vi) a way to avoid interaction between therapeutic exosomes and other cells to assess the safety, feasibility, toxicology, pharmacokinetic and pharmacodynamic characteristics [35]. Thus, the standards for the manufacture, loading efficiency, purification, storage, use, stabilization duration and dosage of exosomes as carriers or their drugs remain a mystery. The International Society for Extracellular Vesicles (ISEV) proposed Minimal Information for Studies of Extracellular Vesicles (“MISEV”) 2018 guidelines which include tables and outlines of suggested protocols and steps to follow for documenting specific EV-associated functional activities [34]. To large-scale production of exosomes for clinical use, several recent studies have developed efficient scalable production protocols. A recent study by Lee et al. shows a reproducible large-scale isolation of exosomes from adipose tissue-derived MSCs and their application in acute kidney injury, reporting on the use of tangential flow filtration (TFF) [239]. Another study develops a medium formulation based on pooled human platelet lysate (pHPL), free from animal-derived xenogenic additives and depleted of EVs to purify exclusively human MSC-derived EVs [240]. With this GMP-grade protocol, Pachler et al. will identify active components in therapeutic EVs for future clinical application. Cha et al. successfully amplified MV secretion from MSCs compared to the conventional culture method using a simple and efficient 3D-bioprocessing method, facilitating diverse applications of MSC-derived MVs from the bench to the bedside [241]. However, the solutions to these issues are awaited with great interest.

Despite the challenges facing the exosome-based drug delivery system, these endogenous vesicles have great potential in the biomedical field. Furthermore, developing artificial exosome mimetics with similar homing properties and low side effects to exosomes could be an interesting concept to pursue to obtain clinical scale production of nanocarriers. In summary, the application of nanotechnology and nanomedicine provides great potential for prevention and treatment of human autoimmune diseases, especially in SLE therapy.

## Figures and Tables

**Table 1:** Studies that analyze the potential of modifying exosomes to deliver therapeutic cargos in reducing kidney inflammation and maintaining joint integrity, which are target areas of systemic lupus erythematosus.

Donor Cell	Therapeutic Cargo	Targeting Peptides	Loading Methods	Effect on Target Cell
MSC^201^	miR-150-5p		Transfection parent cell	Reduction of joint destruction
MSC^200^	miR-146a/miR-155		Transfection parent cell	Enhance anti-inflammatory response
BMSC^202^	miR-192-5p		Transfection parent cell	Delay inflammatory response
MSC^203^	miR-320a		Transfection parent cell	Attenuate bone damage
BMSC^205^	miR-199-3p		Transfection parent cell	Antiapoptotic effect in renal cells
MSC^206^	miR-140-3p		Transfection parent cell	Enhance cartilage regeneration
MSC^207^	miR-92-3p		Transfection parent cell	Inhibition of cartilage degradation
BMSC^208^	miR-210		Transfection parent cell	Enhance chondrocyte proliferation
RAW 264.7^213^	Dexamethasone	FA-PEG-Col	Electroporation	Reduction of inflamed joints
EL-4^121^	Curcumin		Incubation	Enhance anti-inflammatory activity
MSC^219^	siRNA KLF3-AS1		Transfection parent cell	Enhance cartilage repair and chondrogenesis
DC^220^	miR-140	CAP-Lampb2	Electroporation	Inhibition of cartilage degradation
ADMSC^86^	miR-10a		Electroporation	Reduce LPS chondrocyte injury

ADMSC: Adipose derived mesenchymal stem cells; BMSC: Bone marrow derived mesenchymal stem cells; CAP-Lampb2: Chondrocyte-affinity peptide with the Lamp2b; FA-PEG-Col: Folic acidity (FA)-changed polyethylene glycol (PEG)-chitosan oligosaccharide lactate (COL); LPS: Lipopolysaccharide; and MSC: Mesenchymal stem cells.
